# Discovery of Prognostic Biomarker Candidates of Lacunar Infarction by Quantitative Proteomics of Microvesicles Enriched Plasma

**DOI:** 10.1371/journal.pone.0094663

**Published:** 2014-04-21

**Authors:** Arnab Datta, Christopher P. Chen, Siu Kwan Sze

**Affiliations:** 1 School of Biological Sciences, Nanyang Technological University, Singapore, Singapore; 2 Memory, Aging and Cognition Centre, National University Health System, Singapore, Singapore; 3 Department of Pharmacology, Yong Loo Lin School of Medicine, National University of Singapore, Singapore, Singapore; Moffitt Cancer Center, United States of America

## Abstract

**Background:**

Lacunar infarction (LACI) is a subtype of acute ischemic stroke affecting around 25% of all ischemic stroke cases. Despite having an excellent recovery during acute phase, certain LACI patients have poor mid- to long-term prognosis due to the recurrence of vascular events or a decline in cognitive functions. Hence, blood-based biomarkers could be complementary prognostic and research tools.

**Methods and Finding:**

Plasma was collected from forty five patients following a non-disabling LACI along with seventeen matched control subjects. The LACI patients were monitored prospectively for up to five years for the occurrence of adverse outcomes and grouped accordingly (i.e., LACI-no adverse outcome, LACI-recurrent vascular event, and LACI-cognitive decline without any recurrence of vascular events). Microvesicles-enriched fractions isolated from the pooled plasma of four groups were profiled by an iTRAQ-guided discovery approach to quantify the differential proteome. The data have been deposited to the ProteomeXchange with identifier PXD000748. Bioinformatics analysis and data mining revealed up-regulation of brain-specific proteins including myelin basic protein, proteins of coagulation cascade (e.g., fibrinogen alpha chain, fibrinogen beta chain) and focal adhesion (e.g., integrin alpha-IIb, talin-1, and filamin-A) while albumin was down-regulated in both groups of patients with adverse outcome.

**Conclusion:**

This data set may offer important insight into the mechanisms of poor prognosis and provide candidate prognostic biomarkers for validation on larger cohort of individual LACI patients.

## Introduction

Lacunar infarction (LACI) is a subtype of ischemic stroke that accounts for approximately a quarter of all ischemic stroke cases with a higher prevalence in south Asian population [1], [2]. Current stroke guidelines do not differentiate between lacunar and non-lacunar strokes (e.g. large vessel stroke or cardioembolic) with respect to treatment or risk factor modification [1], [3]. Similarly, many of the major secondary stroke prevention trials have not distinguished between different types of ischemic stroke, which may be important in determining the differential protective influence of various therapeutic approaches (e.g., antiplatelet drugs or thrombolysis) [1], [4]. However, mounting evidence suggests differences in LACI pathology in comparison with non-lacunar strokes [1]. Nevertheless, LACI remains a poorly understood area in terms of its etiology, pathophysiology, and more importantly prognosis [2], [5].

Unlike non-lacunar subtypes of ischemic stroke, the short-term prognosis of ischemic small-vessel disease (SVD), including LACI is more favorable with an almost negligible early mortality, an absence of neuropsychological impairment and an excellent neurological recovery. However, LACI causes an increase in the mid- or long-term risk of recurrent vascular events and cognitive impairment or neuropsychological abnormalities. It has been shown recently that the proportion of dementia caused by SVD ranges from 36 to 67% [6]. Therefore, identifying the patient cohorts that are at mid- or long-term risk for recurrent vascular events or secondary complications such as vascular cognitive impairment may allow for improved treatment and prevention paradigms.

Blood-based biomarkers can serve as an alternative tool to complement and improve the prognostic ability of clinical features and neuroimaging. Biomarker for prognosis of ischemic stroke is a relatively new concept compared to biomarkers for diagnosis. No single or panel of blood-based biomarkers has been validated by clinical trials for stroke or related secondary complications. Blood, CSF [7] or brain extracellular fluid [8] has been used as starting materials for biomarker discovery in stroke. Although several studies had been performed to validate protein biomarkers from blood [9], [10], [11], only a few of them were directed specifically to SVD [12], [13], [14], [15]. In addition, most have tried to validate one or a few candidates and although suggested, a proteomics result-guided discovery approach has never been utilized to discover a panel of potential stroke biomarkers [9]. This unbiased systematic approach could be complementary to the traditional hypothesis-driven approach of targeted selection and validation of a single or few proteins.

Plasma microvesicle is a good source of disease biomarkers that entered the circulatory system following their release by cells from various tissues. It has been found that central nervous system (CNS)-specific cell types secrete microvesicles to mediate cell-to-cell communication under physiological and pathological conditions [16], [17], [18], [19]. Here, we hypothesize that the brain cells of LACI patients with poor prognosis under the influence of ischemic stress may release microvesicles into circulation through the compromised blood brain barrier (BBB) during its evolution. Detecting these plasma microvesicles with good sensitivity by downstream proteomics profiling could provide potential biomarkers for LACI prognosis. Isobaric labeling based quantitative proteomics is a popular profiling approach that has found wide application in various areas of science and medicine [20], [21]. Recently, we have successfully combined an iTRAQ-2D-LC-MS/MS-based proteomics strategy as a relative quantitation tool along with various types of biological samples (such as neuroblastoma cell-line, rodent and human brain tissue) to obtain important pathological insights in the area of ischemic stroke [22], [23], [24] and vascular dementia [25]. Here, we apply a similar methodology for comparative profiling of plasma microvesicles from three groups of LACI patients and a group of demographically-matched control to discover potential prognostic biomarkers of LACI. Plasma samples of forty five LACI patients from the European Australasian Stroke Prevention in Reversible Ischemia Trial (ESPRIT) were used for this study [26]. The patients were monitored for up to 5 years after index stroke for adverse outcomes (i.e. recurrent vascular events or decline in cognitive functions). A microvesicle-enriched fraction was obtained by differential centrifugation and ultracentrifugation from the pooled plasma of each group for the iTRAQ experiment.

Analysis of the significantly regulated proteins from the iTRAQ data set revealed an up-regulation of brain-specific myelin basic protein (MBP) apart from proteins related to the integrin signaling [e.g. Integrin alpha-IIb (ITGA2B), Talin-1 (TLN1), and Filamin-A (FLNA)] and coagulation cascade [Fibrinogen alpha chain (FGA), Fibrinogen beta chain (FGB)] that is associated with an unfavorable outcome. Given that blood collection is a simple and cheap procedure; these candidates once validated in larger cohort of LACI patients may have additive value over the existing imaging, clinical or neurobehavioral modalities used in the clinic.

## Materials and Methods

### Reagents

Unless indicated, all reagents were purchased from Sigma-Aldrich (St. Louis, MO, USA).

### Ethics Statement

The study protocol was approved by Singapore General Hospital’s and Nanyang Technological University’s Institutional Review Board and Ethics Committee. Written informed consent was obtained from all patients or legal guardians. The European Australasian Stroke Prevention in Reversible Ischemia Trial (ESPRIT) was registered under http://clinicaltrials.gov with the identifier NCT00161070.

### Sample Collection

The plasma samples were obtained from patients with a nondisabling ischemic stroke who were recruited at the Singapore General Hospital between 1999 and 2005 for the cognitive sub-study of the ESPRIT (ESPRIT-cog). Detailed methodology of ESPRIT and ESPRIT-cog including the exclusion criteria have been reported previously [26], [27]. Briefly, for ESPRIT, patients were eligible if they were within 6 months of a transient ischemic attack (including transient monocular blindness) or a nondisabling ischemic stroke (grade≤3 on the modified Rankin scale [mRS]) of presumed arterial origin [28]. All patients were randomized to either aspirin (100 mg/day) or aspirin combined with dipyridamole (75–450 mg/day). The control plasma was collected from non-stroke subjects at the same site during 2004–2006. EDTA was used as the anti-coagulant during the processing of blood samples. The exclusion criteria were: a possible cardiac source of embolism, high-grade carotid stenosis for which carotid endarterectomy or endovascular treatment was planned, moderate to severe leukoaraiosis on brain imaging (for randomization into anticoagulation), any blood coagulation disorder, any contraindication for aspirin or dipyridamole, and a limited life expectancy [27].

### Neuropsychological Test Battery – Determination of Cognitive Impairment

The cognitive status of the patients was determined by trained research psychologists using standard neuropsychological test battery that has been validated for use in Singapore. Details of the procedure have been described previously [27], [29]. Briefly, the battery assessed 6 domains; 2 memory domains (i.e. Verbal Memory and Visual memory) and 4 non-memory domains (Attention, Language, Visuomotor speed and Visuoconstruction). Failure in at least half of the tests in a domain constituted failure in that domain. Diagnoses of dementia were made according to the DSM-IV criteria [30]. Any patients who were demented at the baseline were excluded from this study. The patients who did not qualify to be demented, included individuals with diagnoses of cognitive impairment no dementia (CIND) –mild (impairment of 1–2 domains), CIND –moderate (impairment of 3–6 domains) and no cognitive impairment (NCI). Diagnoses of dementia and CIND were made after each patient’s baseline and follow up visits.

### Baseline Risk Factors

Risk factor information was collected at baseline. Stroke subtype was classified according to the Oxfordshire Community Stroke Project as total anterior circulation infarct, partial anterior circulation infarct, posterior circulation infarct, or LACI [31]. Vascular risk factor data, such as age, diabetes mellitus status, hypertension, hyperlipidemia, smoking status, ischemic heart disease, peripheral artery disease, as well as past history of stroke, angina and myocardial infarction were obtained verbally from the patient and confirmed with hospital records.

### Experimental Design Guided by Outcome Measures

**Figure 1:**
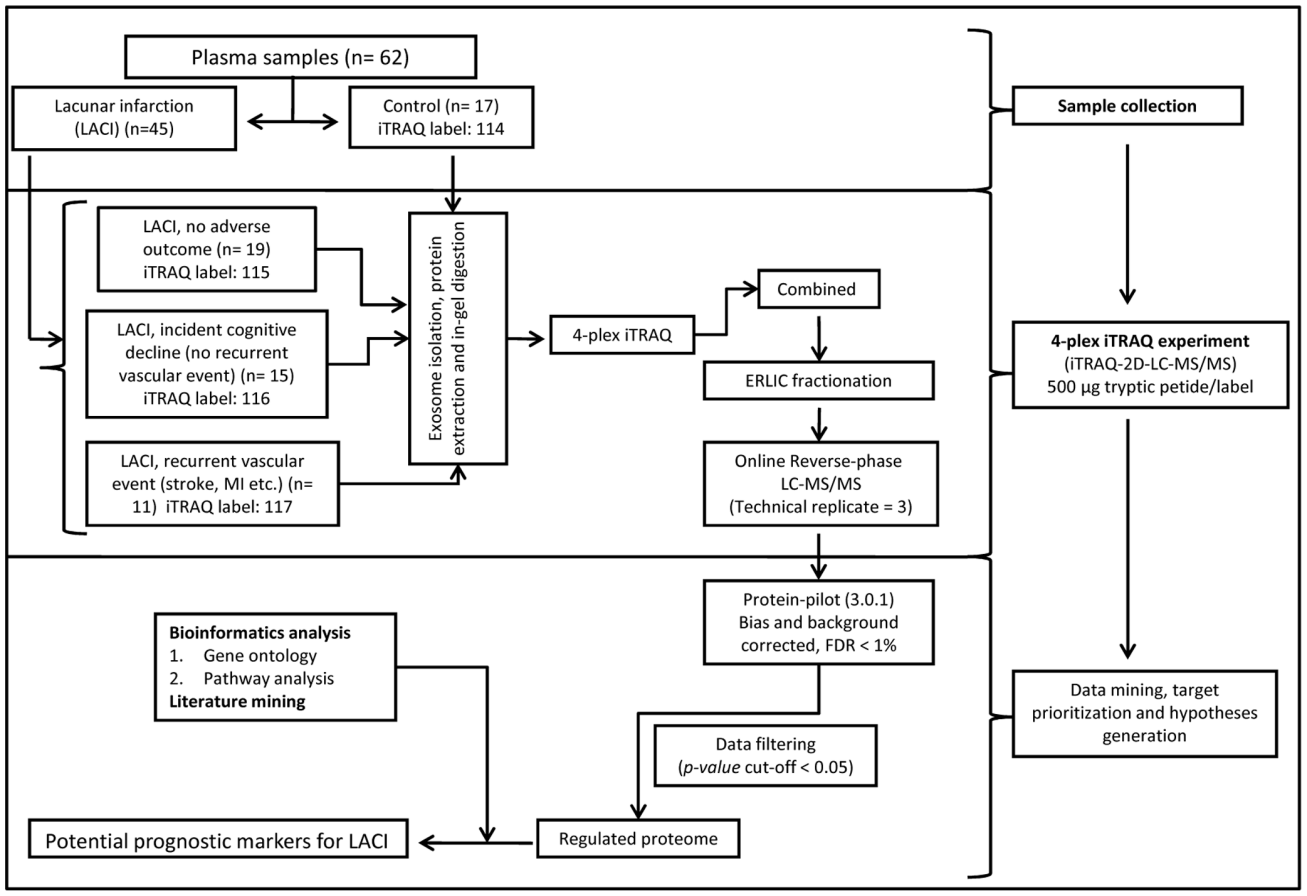
Schematic representation of the experimental design. ERLIC, electrostatic repulsion hydrophilic interaction chromatography.

The experimental design is depicted in Figure 1. The LACI patients were followed up annually for up to 5 years (median follow up 3 years; interquartile range, 2 years) to monitor for the occurrence of any vascular event or for change in the cognitive status. Strokes, peripheral artery disease, intracranial bleeds, and any cardiac ischemia (stable and unstable angina, myocardial infarctions) or deaths from any of the above were considered to be a recurrent vascular event. Any LACI patient having a recurrence of vascular event during the follow-up period was included in the group called “recurrent vascular event” [27], [29]. The patients whose cognitive status declined from the respective baseline status during the course of the prospective study had been assigned to the “cognitive decline” group. Patients who did not suffer a recurrent vascular event or cognitive decline during this period were grouped as “LACI, no adverse outcome”. Accordingly, plasma samples of 45 LACI patients were divided into three groups based on the outcome variables (LACI- no adverse outcome, n = 19; LACI- recurrent vascular events, n = 11; LACI – cognitive decline but no recurrent vascular events, n = 15). The age-matched control group had 17 subjects who never had a stroke or cancer and were cognitively normal at the baseline. The plasma samples were pooled group-wise before processing. A microvesicle-enriched fraction was isolated by sequential centrifugation combined with ultracentrifugation and labeled with isobaric tags that was followed by 2D-LC-MS/MS analysis to improve the depth of identification and quantification. The iTRAQ samples were injected thrice in the LC-MS/MS analysis (technical replicate = 3).

### Sample Preparation

#### Separation of microvesicle-enriched fraction by sequential centrifugation

**Figure 2:**
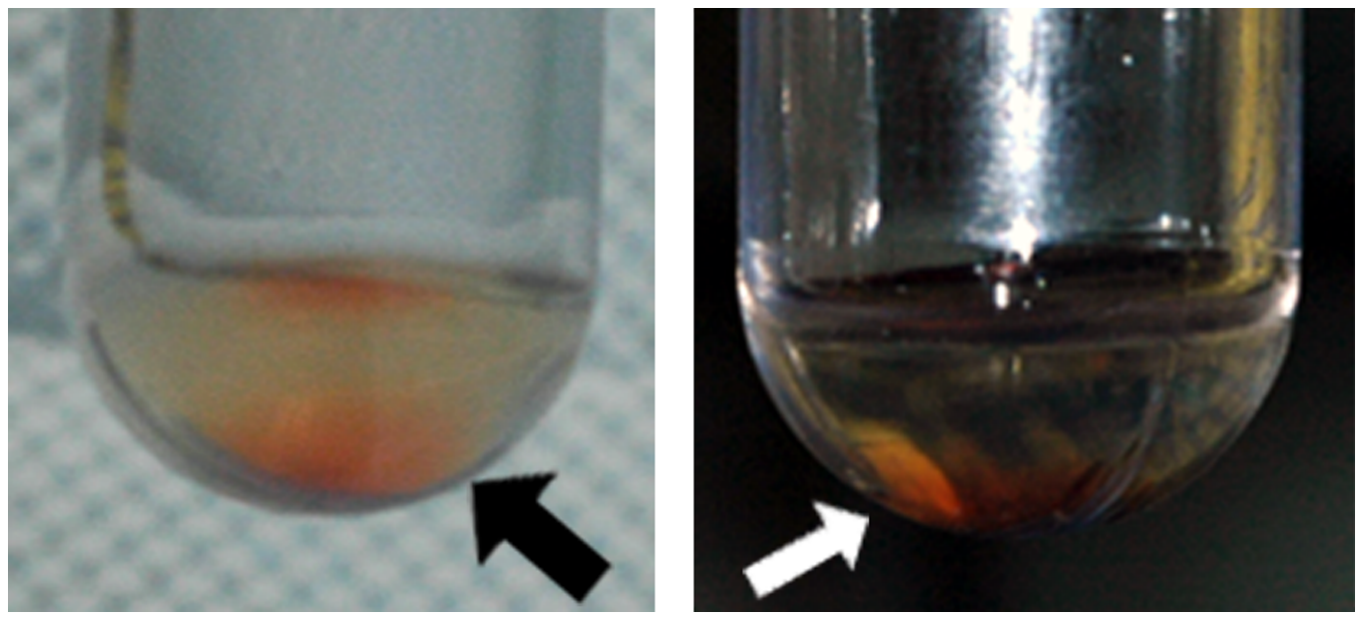
Pictures of a representative microvesicle pellet against different backgrounds. The loose orange-reddish hollow at the bottom of the tubes (shown by arrow) was obtained following ultracentrifugation (200 000 g, 2 h 15 min) of the microparticle-depleted pooled plasma.

Frozen individual plasma samples were thawed on ice and pooled in a group-wise manner to obtain four tubes containing around 5 ml of plasma specimens from each group. The samples were subjected to sequential centrifugation to enrich the microvesicles using a modified protocol as described previously [32], [33]. Briefly, sonicated plasma (5×1 min) was centrifuged at 4,000 g twice for 30 min and then at 12,000 g for 30 min to collect and remove the pellets. The resulting supernatant was subsequently diluted approx. five times with ice-cold 1X PBS before doing ultra-centrifugation at 30,000 g for 2 h to collect the pellet of plasma membrane derived vesicles or microparticles for separate study. The supernatant was ultra-centrifuged again at 200,000 g for 2 h 15 min to collect the microvesicle pellet (Figure 2). The microvesicle pellets were washed at least twice with 1X PBS and were lyophilized. The lyophilisate was dissolved using 50–100 µl of ice-cold dissolution buffer [6% sodium dodecyl sulfate; 20 mM dithiothreitol, 100 mM tris-HCl with Complete Protease Inhibitor Cocktail (COMPLETE, (Roche; Mannheim, Germany)), pH 7.75] by brief vortexing. Protein quantization was performed using 2-D Quant kit (Amersham Biosciences, Piscataway, NJ, USA).

### Proteomics

#### In-Gel tryptic digestion and isobaric labeling

The samples (500 µg/condition) were subjected to denaturing PAGE using a 4%–6%–25% gel following an identical procedure as described previously [23], [24]. Briefly, the diced gel bands were extensively washed with 25 mM TEAB in 50% ACN to completely remove Tris HCl and detergent before reduction and alkylation. They were digested overnight (12.5 ng/µl of sequencing-grade modified trypsin ((Promega, Madison, WI, USA), in 50 mM TEAB, 2% acetonitrile (ACN)) at 37°C. Subsequently, the peptides were extracted and dried before reconstituting them into 0.5 M triethylammonium bicarbonate (TEAB) and labeled with respective isobaric tags of 4-plex iTRAQ Reagent Multi-Plex kit (Applied Biosystems, Foster City, CA, USA) as follows: Control, 114; LACI-no adverse outcome, 115; LACI- recurrent vascular events, 116; LACI – cognitive decline but no recurrent vascular events, 117 (Figure 1).

### Electrostatic Repulsion and Hydrophilic Interaction Chromatography (ERLIC)

The combined iTRAQ sample was desalted by Sep-Pak C18 SPE cartridges (Waters, Milford, MA, USA). A modified ERLIC with volatile salt-containing buffers was adopted [34]. The dried iTRAQ-labeled peptides were reconstituted in 200 µl of Buffer A (10 mM NH_4_HCO_2_, 85% ACN, 0.1% formic acid (FA)) and fractionated using a PolyWAX LP column (200 × 4.6 mm; 5 µm; 300 Å) (PolyLC, Columbia, MD, USA) on a Prominence HPLC system (Shimadzu, Kyoto, Japan) in a 65 min gradient with Buffer B (30% ACN, 0.1% FA). The HPLC gradient was composed of 100% buffer A for 10 min; 0–25% buffer B for 35 min; then 25–100% buffer B for 10 min; followed by 100% buffer B for 10 min. The chromatogram was recorded at 280 nm. Eluted fractions were collected in every 1 min, and then pooled into 34 fractions depending on the peak intensities, before drying them in a vacuum centrifuge. They were stored at −20°C till MS analysis.

### Reverse Phase LC-MS/MS Analysis using QSTAR

The iTRAQ-labeled peptides were reconstituted with 0.1% FA, 3% ACN and analyzed using a HPLC system (Shimadzu) coupled with QSTAR Elite Hybrid MS (Applied Biosystems/MDS-SCIEX) as described previously with minor modifications. Briefly, most of the LC parameters for a 90 min gradient including column configuration, gradient and flow rate were kept constant except the mobile phase A composition (0.1% FA in 3% ACN) and sample injection volume (15 µl/injection). Regarding MS parameters, the precursors with a mass range of 300–1600 m/z and calculated charge of +2 to +5 were selected for the fragmentation. The selected precursor ion was dynamically excluded for 20 s with a 50 mDa mass tolerance. The maximum accumulation time was set at 1.0 s. All other MS parameters were kept identical as reported previously [23].

### Mass Spectrometric Raw Data Analysis

The Analyst QS 2.0 software (Applied Biosystems) was used for the spectral data acquisition. ProteinPilot Software 3.0, Revision Number: 114 732 (Applied Biosystems) was used for the peak list generation, protein identification and quantification against the concatenated target-decoy Uniprot human database (191242 sequences). The false discovery rate (FDR) of peptide identification was set to be less than 1% (FDR = 2.0×decoy_hits/total_hits). Details of the analysis strategy have been described previously [23].

### Bioinformatics Analysis

The bioinformatics analysis was performed using different attributes such as gene ontology (GO), pathway, protein interaction, tissue specificity, keywords or protein domains of DAVID to extract out hidden trends and enrichment of certain groups of proteins. DAVID uses modular enrichment analysis where the term–term/gene–gene relationships are considered for enrichment *p-*value calculation. It calculates the probability of the number of genes in the list that hit a given biology class as compared to pure random chance with the aid of Fisher’s exact test [35]. Open-source GenePattern software (version 3.3.3) was used for clustering the final list of regulated proteins by hierarchical clustering algorithm [36].

### Western Blot (WB) Analyses

WB was performed after SDS-PAGE by probing with anti-ALB primary antibody (albumin, 1∶5000, rabbit polyclonal; Abcam, Cambridge, UK). 20 µg proteins were used for WB. Immunoreactivity was detected using an HRP chemiluminescent substrate reagent kit (Invitrogen, Carlsbad, CA, USA).

### Statistical Analyses

All statistical analyses were performed using SPSS 13.0 for Windows software (SPSS Inc.). One-way ANOVA followed by post hoc Tukey test was used for scale variables such as age. Nonparametric Kruskal-Wallis H Test was used for comparing ordinal variables such as demographic characteristics and baseline rick factors. Statistical significance was accepted at *p*<0.05.

## Results

### Patient Characteristics

The demographic characteristics, baseline risk factors and cognitive classifications of the study population stratified by outcome measures and control group are summarized in Table 1. The average age of the recruited subjects was 61±10 years; 55% were males and 92% were Chinese. Notably, no significant difference was observed between three groups of LACI patients in terms of most of the baseline risk factors except ‘smoking’ (H(2) = 7.276, *p* = 0.026). This reiterates the importance of having a complementary prognostic tool as traditionally used risk factors (e.g. hypertension, diabetes mellitus) fail to predict adverse outcome in LACI patients.

**Table 1:** Demographic Characteristics of the Patient Population Stratified by the Outcome Measures.

Characteristic N (%)	No adverse outcome(N = 19)	Recurrent vascular events(stroke+MI) (N = 11)	Cognitive decline (no recurrentvascular events) (N = 15)	Healthy control (N = 17)
Age, Mean (SD)†	61 (9)	65 (10)	66 (9)	56 (9)
Sex, Male	17 (90)	8 (73)	5 (33)	4 (26)
Ethnicity, Chinese	17 (90)	8 (73)	15 (100)	17 (100)
Diabetes mellitus	7 (37)	2 (18)	7 (47)	6 (35)
Hypertension	11 (58)	9 (82)	12 (80)	10 (59)
Previous stroke	0 (0)	3 (27)	4 (27)	None
Hyperlipidemia	8 (42)	4 (36)	9 (60)	10 (59)
Ever smoker	5 (26)	6 (55)	1 (7)	1 (6)
Previous ischemicheart disease	2 (11)	2 (18)	1 (7)	3 (18)
Previous myocardialinfarction	0 (0)	0 (0)	1 (7)	
Previous angina	2 (11)	2 (18)	0 (0)	
Previous peripheralartery disease	0 (0)	0 (0)	0 (0)	None
Baseline cognitiveclassification				
NCI	13 (68)	2 (18)	8 (53)	None
CIND-mild	4 (21)	6 (55)	6 (40)	
CIND-moderate	3 (16)	3 (27)	1 (7)	

All values are reported as: N(%), where N indicates the number of observations.

† Values are expressed as: Mean (±standard deviation).

### Proteomics

#### Quality control and filtering of iTRAQ data set

The proteins and peptides that are identified and quantified by iTRAQ experiment were exported from ProteinPilot and listed in the Table S1 (Protein Summary) and Table S2 (Peptide Summary). There were 183 proteins with a FDR of 1.1% when a strict cutoff of unused prot-score >3 (>99.9% confidence) was used as the qualification criteria to minimize the false-positive identification of proteins for subsequent data mining. Around 97% of the identified proteins had ≥2 unique peptides having a confidence of >95%. 288, 377 and 458 proteins were identified with unused score ≥2 (>99% confidence), >1.3 (>95% confidence) and >1.0 (>90% confidence) respectively. Here, our result is either comparable [37], or even better [33], [38], [39] than published reports on plasma proteome profiling. Notably, these studies had started with human plasma and used various approaches, such as, depletion of high-abundant plasma proteins [37], [38], [39] or microvesicle enrichment [33] upstream of the proteomics experiment in order to improve the depth of identification.

Next, a cut-off of *p*-value <0.05 was used for filtering the proteins with significant ratios from each condition. There were 17, 33 and 28 proteins for the three ratios (i.e. 115/114, 116/114 and 117/114) respectively with an acceptable *p*-value after excluding the keratins from the list. Of note, this *p*-value is not related to the biological variation as a pooling strategy was adopted during the proteomics sample preparation. The groups with adverse outcome (either recurrent vascular event or cognitive decline) following LACI had higher percentage of perturbed proteins in the plasma microvesicles (33/183 and 28/183) in comparison with the LACI patients with a good recovery profile (17/183). Overall, 43 proteins having at least one ratio with an acceptable level of confidence were shortlisted for the bioinformatics analysis to retrieve useful biological trends (Table 2).

**Table 2:** The List of Qualified and Regulated Proteome from Microvesicle-enriched Plasma *^a.^*

N	Unused	%Cov (95)	AccessionNumber	Protein name	GeneSymbol	Peptides(95%)	115∶114	116∶114	117∶114	GO/pathway
1	1099.8	86.2	P01023	Alpha-2-macroglobulin	A2M	2474	1.00	**1.02**	**1.07**	CCC, EIA, RTW, ECS
3	257.0	68.8	P01024	Complement C3	C3	291	**1.54**	**0.22**	**0.30**	CCC, EIA, RTW, ECS, IIR
4	226.6	83.7	P02768	Serum albumin*	ALB	433	1.16	**0.14**	**0.12**	ECS
7	155.1	47.9	P02671	Fibrinogen alpha chain	FGA	267	0.92	**4.79**	**3.94**	CCC, RTW, ECS
10	124.2	78.0	P02675	Fibrinogen beta chain	FGB	209	**0.29**	**2.09**	**1.20**	CCC, ECS
11	106.0	67.0	P00738	Haptoglobin	HP	171	1.94	1.80	**0.58**	ECS
13	99.4	57.2	P02679	Fibrinogen gamma chain	FGG	193	**0.30**	**1.38**	**0.55**	CCC, RTW, ECS
18	71.1	15.7	P04275	von Willebrand factor	VWF	44	**1.36**	**3.94**	**4.25**	FA, CCC, RTW, ECS
22	52.9	18.1	P01031	Complement C5	C5	29	**1.32**	**0.36**	0.56	CCC, EIA, RTW, ECS, IIR
23	51.9	8.8	Q9Y6R7	IgGFc-binding protein	FCGBP	31	1.02	**1.57**	**1.84**	ECS
25	49.1	41.7	*Q5VVQ8*	Complement component 4 binding protein, alpha	C4BPA	40	1.27	**1.42**	**0.35**	CCC, RTW, ECS, IIR
27	45.3	68.9	P02647	Apolipoprotein A-I	APOA1	30	0.77	**0.33**	**0.52**	LT, ECS
30	41.9	34.5	Q08380	Galectin-3-binding protein*	LGALS3BP	44	**0.30**	**0.26**	0.78	ECS
31	41.0	14.0	*Q60FE2*	Non-muscle myosin heavy polypeptide 9	MYH9	25	0.49	**3.19**	**2.05**	
33	39.8	11.1	P21333	Filamin-A	FLNA	21	0.88	**2.94**	**2.58**	FA, ECS
34	38.6	11.0	Q9Y490	Talin-1	TLN1	19	0.65	**2.61**	**2.11**	FA, ECS
36	37.4	69.7	P69905	Hemoglobin subunit alpha	HBA1	56	**3.87**	**1.89**	0.49	
37	35.2	47.3	O43866	CD5 antigen-like	CD5L	28	**0.46**	0.86	1.04	ECS
40	32.6	38.5	P01009	Alpha-1-antitrypsin	SERPINA1	21	0.75	**0.09**	**0.20**	CCC, EIA, RTW, ECS
42	30.3	22.3	P00747	Plasminogen	PLG	14	**1.43**	0.91	1.14	CCC, RTW, ECS
45	28.5	23.8	*Q4W5C3*	Kallikrein B, plasma (Fletcher factor) 1,isoform CRA_b	KLKB1	17	**0.59**	**0.55**	1.02	CCC, RTW, ECS
48	24.9	20.3	P07225	Vitamin K-dependent protein S	PROS1	13	1.43	1.05	**0.49**	CCC, EIA, RTW, ECS
50	24.0	15.0	*B2RMS9*	Inter-alpha (Globulin) inhibitor H4(Plasma Kallikrein-sensitive glycoprotein)	ITIH4	14	**1.69**	0.73	0.75	EIA, RTW, ECS
51	21.4	29.6	O14791	Apolipoprotein L1	APOL1	15	0.61	**0.14**	0.69	LT, ECS, IIR
52	21.3	14.7	*Q1HP67*	Lipoprotein, Lp(A)	LPA	14	**2.81**	**9.12**	**7.38**	LT, EIA, RTW, ECS
53	19.9	27.9	P02790	Hemopexin	HPX	12	1.18	0.51	**0.30**	ECS
61	16.0	27.8	P02649	Apolipoprotein E	APOE	8	0.69	**0.65**	**0.49**	LT, ECS
67	13.7	8.0	P08514	Integrin alpha-IIb*	ITGA2B	6	1.12	**2.13**	1.42	FA
74	12.5	36.5	P01591	Immunoglobulin J chain	IGJ	16	0.77	**1.96**	**2.75**	ECS
76	12.2	20.6	P27169	Serum paraoxonase/arylesterase 1	PON1	6	0.75	**0.41**	**0.23**	ECS
80	10.7	21.4	P02686	Myelin basic protein	MBP	5	1.08	**14.19**	**8.87**	
84	10.2	18.3	*Q5UGI6*	Serine/cysteine proteinase inhibitor cladeG member 1 splice variant 2 (Fragment)	SERPING1	5	1.10	0.70	**0.41**	CCC, EIA, ECS, IIR
115	6.4	20.0	P02652	Apolipoprotein A-II	APOA2	2	0.61	**0.48**	0.65	LT, EIA, RTW, ECS
15	76.0	42.2	*A8K5A4*	cDNA FLJ76826, highly similar to Homo sapiensceruloplasmin (ferroxidase) (CP), mRNA		63	**1.74**	**0.13**	0.94	
24	49.5	33.1	*B2R950*	cDNA, FLJ94213, highly similar to Homo sapienspregnancy-zone protein (PZP), mRNA		233	0.90	**0.43**	1.27	
26	47.1	44.5	*A8K5J8*	cDNA FLJ75066, highly similar to Homo sapienscomplement component 1, r subcomponent(C1R), mRNA		31	**0.67**	**0.25**	**0.23**	
41	31.4	10.2	*B4E1Z4*	cDNA FLJ55673, highly similar to Complementfactor B (EC 3.4.21.47)		13	**1.41**	**0.41**	**0.44**	
56	18.2	26.4	*Q8WVW5*	Putative uncharacterized protein (Fragment)		9	**0.09**	1.71	1.16	FA, RTW
57	18.2	8.7	*Q59E99*	Thrombospondin 1 variant (Fragment)		8	**0.55**	1.17	1.03	FA, ECS
70	13.4	16.1	*B3KS79*	cDNA FLJ35730 fis, clone TESTI2003131, highlysimilar to ALPHA-1-ANTICHYMOTRYPSIN		7	1.01	0.51	**0.38**	
75	12.2	9.6	*B7Z550*	cDNA FLJ59731, highly similar to Complement component C8 beta chain	C8B	6	0.83	**0.44**	**0.39**	
145	4.6	14.6	*A8K486*	Peptidyl-prolyl cis-trans isomerase*		2	1.96	**7.05**	**5.45**	
176	3.3	2.2	*Q59GB4*	Dihydropyrimidinase-like 2 variant (Fragment)		1	1.38	**5.40**	3.84	

a The list contains quantitative information of the selected proteins from bias and background corrected iTRAQ data set. The denominator is the demographically matched control. Unused and %coverage are parameters related to the confident identification of proteins. This list contains 43 candidates qualified (out of 183) through the initial filters [i.e., unused prot score >3.0 and FDR = 1.1% (confident identification), *p*-value <0.05 (significantly different from 1) for at least one ratio]. The Significant ratios are indicated in bold. The uniport accession numbers of the ‘unreviewed’ proteins are indicated in *italics* form. The protein whose evidence is available only at the level of transcript is not provided with a gene symbol. The last column provides information about GO or pathway. CCC = complement and coagulation cascade, ECS = extracellular space, EIA = enzyme inhibitor activity, FA = focal adhesion, IIR = innate immune response, LT =  lipid transport, RTW = response to wounding. *Reported as microvesicle or exosome marker by independent studies.

#### Bioinformatics analysis of perturbed proteome of microvesicle-enriched plasma

Uniprot accession numbers of the shortlisted 43 proteins was uploaded in DAVID to compare them with the human proteome which was used as the background. To check the enrichment, *p*-value≤0.01 and FDR <1% was used as a cut-off. The GO analysis in the ‘biological process’ category shortlisted ‘response to wounding’, ‘acute inflammatory response’ and ‘lipid transport’ as some of the perturbed processes whereas ‘enzyme (peptidase and endopeptidase) inhibitor activity’ was the key ‘molecular function’ that was enriched in the perturbed proteome. A complementary trend was observed for significantly enriched ‘cellular component’ as ‘extracellular region or space’ and ‘platelet alpha granule’ were shortlisted. Searching for enriched pathways using various modules (e.g. KEGG, Biocarta, Reactome) showed ‘complement and coagulation cascades’, ‘intrinsic prothrombin activation pathway’ or ‘integrin cell surface interactions’ as significantly over-represented (Table 2).

**Figure 3:**
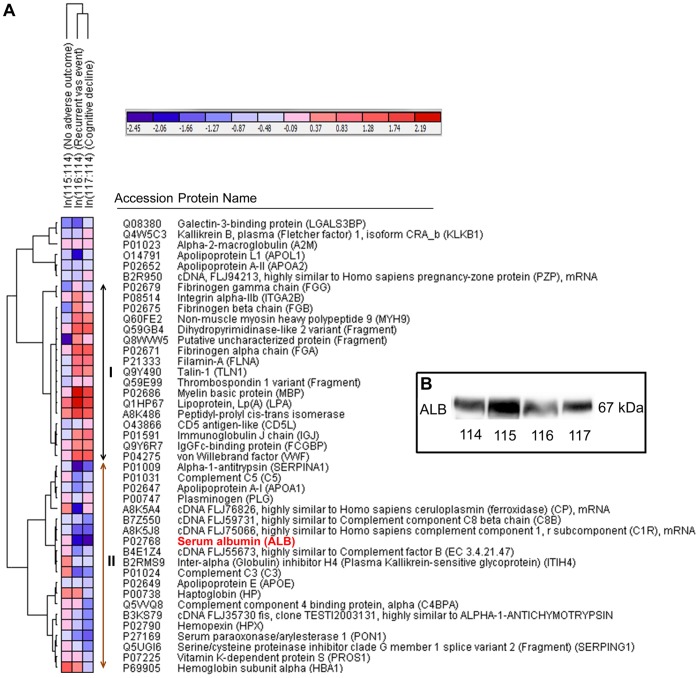
A) Hierarchical clustering of the filtered list of proteins from microvesicle-enriched plasma. Log_2_-transformed ratios (e.g. ln (115/114)) of each protein (row) were presented for all conditions (column). Pearson correlation was applied for the measurement of row and column distance. Globally normalized view was presented here. The color scale of the heat map ranges from saturated blue (value, −2.45) to saturated red (value, 2.19) in the natural logarithmic scale. The proteins were mainly clustered into two parts as shown by I (up-regulated in adverse outcome groups) and II (down-regulated in the adverse outcome groups). The pattern of regulation was similar between recurrent vascular event and cognitive decline group amid subtle differences in magnitudes. MBP and ALB were the two most regulated proteins. The protein names and accession numbers were taken from the uniprot protein database. The gene symbols are provided within brackets along with the protein name, wherever available. B) Technical validation of iTRAQ result by WB analysis of ALB on pooled lysates. ALB showed down-regulation in both LACI groups with adverse outcome, which is consistent with the iTRAQ result.

**Figure 4:**
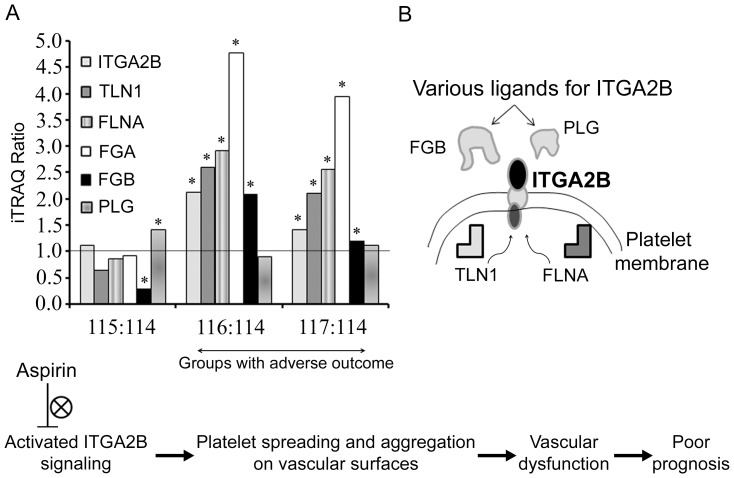
A) Histogram showing the iTRAQ ratios of selected proteins related to focal adhesion (ITGA2B, TLN1 and FLNA) and coagulation cascade (FGA, FGB and PLG). Demographically matched control group was used as the common denominator (i.e. 114) for comparing the three groups of LACI patients. The solid line indicates no change in regulation. The LACI groups with adverse outcome (recurrent vascular event, 116; cognitive decline, 117) had a differential signature in comparison with the LACI group with no adverse outcome (i.e. 115). Up-regulation of proteins related to focal adhesion and coagulation (FGA, FGB) at the baseline is predictive of poor outcome. *Denotes ratios with significant *p*-value (<0.05). B) Schematic diagram showing the interaction of various ligands of ITGA2B on platelet membrane and the probable involvement down-stream intracellular proteins (e.g. TLN1 and FLNA) in vascular dysfunction that may be responsible for poor prognosis. Aspirin partially blocks the integrin signaling as seen in the flowchart.

The hierarchical clustering analysis classified the proteins into two major clusters (Figure 3A, I and II) separating up- and down-regulated proteins in adverse outcome groups. A few trends were apparent. First, the pattern of regulation of the significantly perturbed proteins in both groups with adverse outcome (recurrent vascular events and cognitive decline) was similar in most cases (except Fibrinogen gamma chain and Complement component 4 binding protein, alpha) amid differences in magnitudes only. However, the extent of deregulation is generally more for the ‘recurrent vascular event’ group compared to the ‘cognitive decline’ group (Table 2, Figure 4). This could indicate the involvement of vascular abnormality in both groups which may remain at a subclinical stage in the patients with cognitive decline. Proteins related to ‘enzyme inhibitor activity’ (e.g. Complement C5, Complement C3, Vitamin K-dependent protein S and Inter-alpha (Globulin) inhibitor H4 (ITIH4)) were generally up-regulated in LACI group with better outcome and down-regulated in LACI groups with adverse outcome. A recent study reported the reduction of serum ITIH4 during the first 2–4 days after stroke onset compared to control serum that returned to baseline levels subsequently with the improvement of patients’ condition [40]. Interestingly, proteins related to ‘integrin cell surface interactions’ (e.g. ITGA2B, TLN1, FGB and FGA) were down-regulated in LACI patients with no adverse outcome while up-regulated in both groups with adverse outcome. In contrast, the lipoproteins did not show a differential regulation between groups. Most of the lipoproteins (Apolipoprotein E, A-I, A-II and L1) were down-regulated except Lipoprotein, Lp(A) which was significantly up-regulated across the LACI groups in comparison with the control. Overall, ALB and MBP were the two most deregulated candidates. The abundance of ALB was validated using pooled samples with WB analysis to check the technical reliability of the iTRAQ result. The WB result showed consistent trends with the iTRAQ result (Figure 3B).

## Discussion

Here we report the significantly altered plasma proteome of microvesicle-rich fraction by comparative profiling of three groups of prospectively followed-up LACI patients and a demographically matched control group that could predict adverse outcome in the surviving LACI patients.

### Quantitative Proteomics of Microvesicle-enriched Fraction – An Alternative Approach of Biomarker Discovery for LACI Prognosis

The proteomics approach for biomarker discovery from crude plasma is technically limited by its complexity and extreme dynamic range (>10^10^), thereby resulting in poor sensitivity for detecting low abundant plasma proteins [41]. To overcome this challenge, multiple approaches have been described that includes biophysical fractionation, enrichment of target sub-proteome and immunodepletion of the abundant interfering proteins. However, none of them were able to significantly outclass the other techniques [38], [42]. Here we adopted an alternative approach of targeting the plasma microvesicles in order to enrich the low abundant pathogenic proteins for quantitative profiling. Microvesicles, including exosomes are membrane-bound particles and are increasingly being recognized as reservoirs of potential biomarkers [43]. They are reported to be involved in the pathogenesis of various diseases such as ischemic stroke, thrombosis, diabetes, inflammation, atherosclerosis and vascular cell proliferation [18], [44], [45]. Microvesicles can be secreted from endothelial, circulatory (e.g. platelets, leukocytes, erythrocytes), and even central nervous system (CNS)-specific cell types (e.g. microglia and oligodendrocytes) [16], [17], [18].

The discovery of circulatory biomarkers for neurological disorders represents an additional challenge as brain parenchyma remains selectively accessible by the systemic circulation due to the presence of BBB under the physiological condition. This makes blood an indirect reflector to sense any events happening inside the brain tissue. However, different cell types of the brain (e.g. microglia and oligodendrocytes) are reported to release microvesicles for delivering signals to the neighboring cells and external environment [18]. A fraction of these microvesicles may drain into the cerebrospinal fluid (CSF) or eventually in the blood. In addition, ischemic SVD is well-known to cause an endothelial dysfunction and a diffuse increase in the BBB permeability that may facilitate the leakage of microvesicles in the general circulation [46]. Hence, profiling of circulatory microvesicle-enriched fractions by quantitative proteomics during the post-stroke recovery phase constituted a technically and conceptually preferred strategy to investigate the on-going neuro-pathological processes and to discover useful prognostic markers. Accordingly, the detection of several commonly known exosome markers (e.g. Galectin-3-binding protein, ITGA2B, Peptidyl-prolyl cis-trans isomerase) in the shortlist of perturbed candidates as well as in the complete list (e.g. CD9, CD81, Gelsolin, Glyceraldehyde-3-phosphate dehydrogenase, Pyruvate kinase, Tubulin alpha-1B and beta-1) of confidently identified proteins indicated the successful enrichment of plasma microvesicles, including exosomes for this study (Table 2, Table S1) [43].

Blood-based biomarker studies in the area of ischemic stroke mostly correlated acute levels of biomarkers (within first week after stroke onset) to short term outcome (e.g. death, disability or infarct volume) without focusing on certain subtypes of ischemic stroke. Most of the investigational biomarkers are proteins of extra-cranial source that are related to inflammation, cardiovascular system and hemostasis apart from few proteins of brain origin [47], [48]. Our study, using the microvesicle profiling approach, has identified differentially regulated peripheral as well as brain-specific candidates (e.g. MBP and glial fibrillary acidic protein(GFAP)) targeting only lacunar stroke while relating them to the long-term outcome measures such as cognitive decline and recurrent vascular events (Table 2, Table S1). In addition, the plasma was collected during the convalescent phase following the index event thus effectively evading the acute systemic response.

### Up-regulation of Integrin Signaling – Probable Failure of Aspirin Therapy

The down-regulation of candidates from the coagulation cascade (e.g. FGB) and integrin signaling pathway (e.g. ITGA2B, FLNA and TLN1) and up-regulation of plasminogen (PLG) in the LACI patients was associated with no adverse outcome. PLG is secreted as a zymogen and activated by proteolysis through tissue plasminogen activator to generate plasmin, which dissolves fibrin in blood clots and helps to restore circulation. ITGA2B or CD41 is a platelet membrane glycoprotein and receptor for diverse ligands including fibronectin, fibrinogen, plasminogen, prothrombin, and thrombospondin. Activated ITGA2B mediates platelet spreading and aggregation on vascular surfaces during hemostasis and thrombosis. It has been shown that TLN1 can independently activate β integrin by binding on its cytoplasmic tail [49] (Figure 4). FLNA on the other hand can compete with TLN1 for binding to integrins, thereby regulating its activation under certain circumstances [50]. In a recent study, involvement of TLN1-dependent activation of ITGA2B or Rac1 in platelets has been demonstrated for late phase stability of thrombus on undisrupted endothelial cells [51]. Thus, the overall suppression of integrin signaling in patients with no adverse outcome is complementary to the down-regulation of its ligands (i.e. FGB) and the up-regulation of PLG. Aspirin has been reported to partially inhibit the inside-out ITGA2B signaling apart from its anti-platelet action [52]. As all LACI patients were on aspirin therapy, down-regulation of the pro-aggregatory platelet proteins and suppression of proteins from integrin signaling pathway in platelet should be related to the desired anti-thrombotic effect (Figure 4). This speculation was further validated as all the above-mentioned proteins (i.e. ITGA2B, TLN1, FLNA, FGA, FGB, and PLG) showed opposite or no regulation in both groups with adverse outcome (Figure 4A). High plasma fibrinogen is well-studied to be an independent risk factor for stroke and is associated with an increased risk of recurrent cardiovascular events, when stroke sub-types were not specified [53]. In another study dealing with SVD in particular, a positive correlation was obtained between fibrinogen level and the amount of leukoaraiosis [54]. Fibrinogen is one of the main determinants of plasma viscosity. Thus, higher levels of fibrinogen in surviving LACI patients may aggravate the cerebrovascular dysfunction through hemorheologic impairment or by inducing a state of hypercoagulability [54].

### Up-regulation of Brain-specific MBP – Predictor of Poor Outcome

Brain-specific MBP has been detected in the systemic circulation in nanogram concentration during the acute phase (e.g. hrs to few days) of ischemic stroke and are correlated with acute (24 hrs) or subacute (3 months) outcomes using targeted assays [55], [56]. Here, we confidently identified MBP with five unique peptides (unused score = 10.7) by a proteomics profiling approach justifying the utility of this methodology for sensitive detection of low abundant plasma proteins. Our result indicates that significantly higher MBP concentration during convalescent stage is associated with adverse outcome which is consistent with the previous reports. BBB abnormality is generally more diffuse in small vessel stroke compared to non-lacunar stroke subtypes that may cause gradual and sustained leakage of brain-specific MBP into general circulation [46]. Chronic hypoperfusion of the white matter leading to progressive and selective death of oligodendrocytes by apoptosis and subsequent degeneration of myelinated fibres have been demonstrated in animal models of SVD [57]. Hence, the release of MBP, which is a structural component of CNS myelin and a marker of oligodendrocyte, either signifies an increased glial injury or an increased permeability of the BBB. Both of these may be responsible for the recurrent vascular event or cognitive decline in the groups with adverse outcome. Leaked MBP along with other CNS specific proteins may also act as antigenic signals to activate systemic immune response that could exacerbate the ischemic injury through inflammatory pathways [58].

### Down-regulation of ALB – Indicative of Poor Outcome

Our result showed that significant down-regulation of plasma ALB is associated with adverse outcome among the surviving LACI patients (Table 2, Figure 3B). Several clinical studies have reported higher concentration of circulatory ALB at admission, which is predictive of a better functional outcome and lower mortality in ischemic stroke patients [59], [60]. However, unlike others, we have seen a complementary trend by focusing on patients with non-disabling lacunar stroke only. ALB is known to be neuroprotective in preclinical animal models of stroke and was under clinical trial as a potential neuroprotective agent [61], [62]. It might play a beneficial role through its antioxidant, prothrombolytic action or by promoting and sustaining perfusion in the cerebral microcirculation [63]. Hence, a procoagulatory condition as discussed previously is complementary with the down-regulation of ALB in the groups with poor outcome.

## Limitations

The samples had been stored for more than 5 yrs (up to maximum 12 yrs) at −80°C, which should be taken into account before comparing the data with similar studies during a meta-analysis. However, it should be kept in mind that being a study to discover potential prognostic biomarkers while targeting long-term outcome variables, major part of the waiting time is included in the study duration. Further, similar storage time is a common occurrence in biomarker studies and shown to adequately preserve the quality of frozen samples when compared with freshly collected specimens for various circulatory proteins such as insulin-like growth factor-I and transforming growth factor β [55], [64].

The microvesicles obtained by sequential centrifugation and ultracentrifugation are often contaminated by co-sedimenting vesicles and protein aggregates. We have acknowledged this by describing the fraction as ‘microvesicle-enriched’ instead of a pure microvesicle preparation. However, there is no optimized and universally-accepted protocol for isolating pure microvesicles. The source of the plasma microvesicles is also unknown in our study, which may either be derived from circulatory or even some cells of CNS origin as seen by the detection of MBP and GFAP as some of the identified candidates. The traditionally used flow cytometric approaches may also fail to detect microvesicles of specific source due to their ultra-low abundance [18]. Hence, the results from this microvesicle-enriched preparation having an undefined anatomical origin should be interpreted with caution.

The small amount of the starting plasma (∼ 5 ml after pooling) and low yield of the resulting microvesicles did not allow extensive validation of the candidate makers on pooled or individual samples. Hence, no conclusion could have been drawn about the biological variation of candidate biomarkers. Validation using multiple reaction monitoring based mass spectrometric approach or higher volume of starting samples could alleviate this problem.

## Concluding Remarks

Our study is the first of its kind where a discovery proteomics approach was used to identify prognostic circulatory biomarkers of ischemic stroke. The therapeutic importance of plasma microvesicle and the technical advantage of the iTRAQ quantitative proteomics are addressed in a unique experimental design. We also proposed several hypotheses for further testing in bigger population of individual patients. The up-regulation of many platelet related proteins including the proteins of integrin pathway and coagulation cascade probably due to the failure of an anti-platelet therapy is associated with the adverse outcome in the LACI patients. Reverse regulation of MBP and ALB could indicate underlying pathological changes ongoing during the convalescent stage of LACI.

As the plasma levels of many of these candidates are modifiable via drugs or changes in lifestyle, the perturbed candidates alone or ‘as a panel’ can be tested to stratify the high-risk group of patients on priority for hospital admission, treatment or rehabilitation or to monitor the effect of therapy on the long-term functional outcome of the disease. Conversely, these proteins can also be used as surrogate markers in LACI related clinical trials to monitor the consequences of therapeutic interventions [65]. Our study will facilitate a better understanding of the underlying pathology and stimulate research interest on individual candidates because it is difficult to acquire comparable data set from LACI affected brain samples. In conclusion, this study will foster the emerging area of quantitative clinical proteomics as a viable tool for the discovery of novel biomarkers for ischemic stroke.

## Raw Data Availability

The mass spectrometry proteomics data have been deposited to the ProteomeXchange Consortium (http://proteomecentral.proteomexchange.org) via the PRIDE partner repository [66] with the dataset identifier PXD000748.

## Supporting Information

Table S1 **Complete information of the full list of the qualified proteins obtained from the bias and background corrected iTRAQ data set.**
(XLSX)

Table S2 **Full list of the identified peptides exported from the ProteinPilot software obtained following database searching.**
(XLSX)
